# A curated bacterial and archaeal 16S rRNA Gene Oral Sequences dataset

**DOI:** 10.1038/s41597-025-05050-4

**Published:** 2025-05-02

**Authors:** Lara Vázquez-González, Alba Regueira-Iglesias, Carlos Balsa-Castro, Inmaculada Tomás, María J. Carreira

**Affiliations:** 1 Centro Singular de Investigación en Tecnoloxías Intelixentes (CiTIUS), Universidade de Santiago de Compostela, Rúa de Jenaro de la Fuente Domínguez, E15782 Santiago de Compostela, Spain; 2 Instituto de Investigación Sanitaria de Santiago de Compostela (IDIS), E15706 Santiago de Compostela, Spain; 3 Oral Sciences Research Group, Special Needs Unit, Department of Surgery and Medical Surgical Specialities, School of Medicine and Dentistry, Universidade de Santiago de Compostela, E15782 Santiago de Compostela, Spain; 4 Departamento de Electrónica e Computación, Escola Técnica Superior de Enxeñaría, Universidade de Santiago de Compostela, E15782 Santiago de Compostela, Spain

**Keywords:** Computational biology and bioinformatics, Microbiology

## Abstract

In a given species, genomes and 16S rRNA gene sequences, along with their intragenomic copy numbers, can vary greatly across environments. The gene copy numbers are crucial for technologies which estimate microbial abundances based on gene counts, such as polymerase chain reaction and high-throughput sequencing. In these, taxa with fewer genes may be underestimated, while those with more genes might be overestimated. Therefore, it is essential to have accurate gene copy number databases specific to the niche under study. The 16S rRNA Gene Oral Sequences dataset (16SGOSeq) contains the number of 16S rRNA genes and their variants in the complete genomes of the bacterial and archaeal species present in the human oral cavity. It includes 3,192 complete genomes of oral bacteria and 191 complete genomes of oral archaea, from which the 16S rRNA gene sequences were extracted, and the sequence variants were identified. This oral-specific dataset of prokaryotic organisms and the pipeline followed for its construction can be applied by clinical microbiologists, bioinformaticians, or microbial ecologists in future microbiome research.

## Background & Summary

The oral microbiome is the most diverse and second largest in the human body, with over 700 microbial species detected in the mouth at any time^1^. Of these, an individual’s mouth usually harbours between 200 and 300 predominant bacterial species^2^. The dysbiosis of this community, or imbalance, is a key factor in the onset and development of two of the most widespread diseases worldwide: dental caries and periodontitis^3^. Moreover, alterations in the mouth microbiome have been associated with highly relevant systemic pathologies such as diabetes and cardiovascular diseases^4^.

A range of microbiological techniques have been employed to investigate the oral microbial communities associated with health and disease. Among the most prevalent techniques are polymerase chain reaction (PCR), conventional or quantitative (qPCR), and high-throughput sequencing (HTS). These techniques are typically employed by targeting the 16S ribosomal RNA (rRNA) gene^5,6^. The gene in question comprises approximately 1,500 base pairs (bps) and it is regarded as a reliable phylogenetic marker due to several reasons. Primarily, this gene is ubiquitous in bacteria and archaea, exhibiting relative stability in combining conserved (C) and hypervariable (V) regions. Additionally, complete and easily accessible databases exist^7^.

Nevertheless, utilising the 16S rRNA gene also presents certain limitations. One of the most significant is the intragenomic redundancy, which refers to the presence of more than one identical or distinct genes per genome^8–10^. Under the concept of genetic variants, it could be considered the sequences of genes in a genome that differ in at least one nucleotide. Therefore, a variant can have multiple copies per genome. Technologies such as qPCR and HTS are employed to estimate microbial abundance based on gene counts. Consequently, the outcomes derived from these may be influenced to the extent that taxa with a low number of genes may be underestimated, while those with high numbers may be overestimated^10^. Furthermore, heterogeneity between the multiple gene copies within a genome may result in overestimating and taxonomic misassignments^9^.

There are several methodologies for correcting the variation in intragenomic gene copy numbers, such as CopyRighter^11^ or PICRUSt^12^. However, these methodologies are characterised by significant drawbacks. Primarily, these methods are highly dependent on the database used, and therefore, inaccurate estimates may result from incomplete or erroneous data^13^.

The Ribosomal RNA Operon Copy Number Database (rrnDB)^14^ and RiboGrove^15^ provide information on the number of 16S rRNA genes in the genomes of bacteria and archaea. However, the genomes and 16S rRNA gene sequences, as well as their intragenomic copy number, in a given species may vary among environments or niches^16,17^. This variability may be attributed to adaptation to different environmental conditions, selective pressures, or evolutionary events such as gene acquisition through horizontal transfer. It is observed that the greater the pressure or difference between environments, the greater the variability^17–20^. Furthermore, neither of the above databases allows for the identification of the different intragenomic gene variants^14,15^.

On the other hand, the rrnDB^14^ does not allow the selection of sequences from specific taxonomic groups or taxa; the complete database must be downloaded, and subsequent manual selection must be performed. Moreover, the developers of rrnDB^14^ claim to perform quality control of the sequences using data from the Ribosomal Database Project (RDP)^21^. The use of phylogenetically diverse databases can produce classification errors because they contain taxonomically misannotated 16S rRNA gene sequences (i.e., annotation rates in the RDP are approximately 10%)^22^. They also represent differently the environments included, varying substantially in the quality of the classifications^23^.

Preference for the use of environment-specific databases has been demonstrated not only in the oral cavity^24^ by focusing on the expanded Human Oral Microbiome Database (eHOMD)^25^, but also on the vaginal^26^, bovine^27^, bee^28^, dairy products^29^, and wastewater treatment plants microbiomes^30^. Overall, these studies have proven that the use of niche-specific databases improves the accuracy of taxonomic classifications by aligning reference sequences more closely with the microbial communities under investigation, and reduces the number of unassigned reads.

As a last remark, it is notable that numerous examples of species from the same genus have been associated with opposite mouth conditions^31,32^. Consequently, it is recommended that oral microbiology analysis be performed at the lowest taxonomic level possible.

In light of the aforementioned considerations, the objective of this study was to develop a curated dataset comprising all 16S rRNA gene sequences present in the genomes with complete sequencing status of bacterial and archaeal species inhabiting the human mouth. The oral prokaryote-specific dataset, designated 16SGOSeq (16S rRNA Gene Oral Sequences dataset), contains information regarding the size of complete genomes and the size and number of genes per genome and gene variants per genome for taxonomic categories from domain to strain level. All gene sequences are designated at the lowest possible taxonomic level, and users can filter by the desired taxonomic level/taxon and calculate data averages.

## Methods

The 16SGOSeq dataset is a curated sequence dataset based on a collection of genetic sequences that have undergone a systematic and rigorous process of selection, validation, classification and updating, thus guaranteeing their high quality, accuracy and reliability for subsequent scientific uses^33^.

**Fig. 1:**
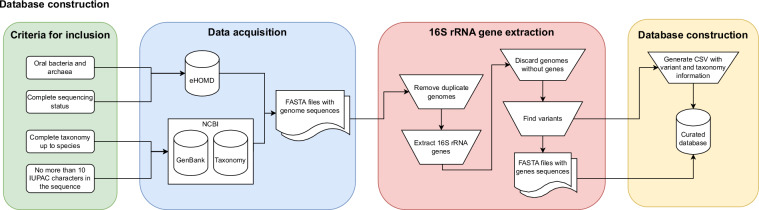
Process flow diagram describing the construction and curation of the 16SGOSeq dataset.

This dataset was constructed following a number of criteria regarding genome inclusion and the curation procedures were employed for the sequences, as illustrated in Fig. 1.

### Data acquisition. Obtaining complete oral bacterial and archaeal genomes

Firstly, the inclusion criteria for the bacterial and archaeal genomes were set to ensure the sequences’ quality. The following criteria were thus established:

1. The bacterial and archaeal taxa were limited to those identified as inhabitants of the oral cavity.
2. Only genomes with complete sequencing status according to the expanded Human Oral Microbiome Database (eHOMD)^25^ were included.
3. The genomes included in this study are those with complete taxonomy up to the species level (i.e., they had to have a specific name for the domain, phylum, class, order, family, genus, and species). Those with ambiguous or disputed taxonomies were eligible (e.g. *Candidatus* taxa). Those with the “unclassified” term at any level were not eligible.
4. Genomes with no more than 10 consecutive ambiguous International Union of Pure and Applied Chemistry (IUPAC) nucleotides were included.

Consequently, the sequences and other information that met the inclusion criteria were downloaded. All available data on bacterial taxa within the mouth was acquired from the publicly available eHOMD oral-specific database^25^ (https://www.homd.org/ftp//genomes/NCBI/V10.1). Only genomes with complete sequencing status according to the eHOMD were chosen, resulting in a total of 3, 128 complete genomes out of the 8, 622 on the eHOMD website. Each genome was assigned one or more GenBank identifiers, which were included in the downloaded genome’s file as different sequences. In the majority of cases, the identifiers corresponded to chromosomal DNA. However, in some instances, they also corresponded to plasmids. Both were considered in the completion of the dataset.

Regarding the oral archaea, the eHOMD^25^ only encompasses complete genome information for a single species, namely *Methanobrevibacter oralis*. For this reason, we used an initial list obtained as part of a previous investigation^34^, which comprised 177 different archaea found in the human mouth and their corresponding GenBank identifiers^35^ to obtain complete publicly available archaeal genomes from the NCBI database^36^. This archaeal list is shown in Supplementary Table S1 of the Supplementary Information. The provenance of these complete genomes was: environmental (91%), human niches (3%), and others (6%).

The Entrez Programming Utilities (E-utilities)^37^ tool was employed to retrieve pertinent data from the diverse NCBI databases, including the Taxonomy^38^ and GenBank^35^ databases. The Entrez module from the BioPython^39^ package was employed in a Python script (version 3.9.0, http://www.python.org/) to facilitate the transmission of requests to the databases and the acquisition of the oral-archaeal genomes and their corresponding taxonomies, as well as the oral-bacterial metadata.

Genomes that lacked a complete taxonomic classification up to the species level were excluded from the analysis. Furthermore, some genomes included non-specific nucleotides, which were identified by the IUPAC codes for ambiguous characters. It should be noted that these ambiguous characters or nucleotides may represent two, three, or four possible nucleic acid states^40^, instead of a unique specification for the four nitrogenous bases of the DNA (A, adenine; G, guanine; C, cytosine; T, thymine). It was, therefore, necessary to exclude from the analysis genomes that had more than ten consecutive ambiguous IUPAC nucleotides.

Following the application of the aforementioned criteria, a total of 3, 079 complete genomes of oral bacteria were identified. Of these, several had one or more sequences corresponding to either genomes, chromosomes, or plasmids. Each was evaluated as a complete genome, resulting in a final number of 5, 755 oral-bacterial complete genomes being considered for analysis. These genomes all had the taxonomy complete up to the strain level. A total of 177 complete archaeal genomes were listed, of which 166 had one chromosome, 10 had two chromosomes, and one had five chromosomes. Consequently, a final number of 191 oral-archaeal complete genomes were considered for creating the 16SGOSeq dataset.

### Detection and extraction of 16S rRNA genes

A Python script was developed to extract the 16S rRNA genes. This script utilised a freely available module, search_16S_py^41^, which implements Edgar’s algorithm^42^. The algorithm employs a search strategy that identifies sections of genomes exhibiting a high frequency of 13-mers, which are characteristic of known 16S rRNA genes. Subsequently, the search is conducted within each segment for conserved motifs situated in close proximity to the beginning and end of the gene. The presence of a pair of motifs within the expected length range indicates the presence of the gene and provides consistent and homologous endpoints.

The application of this algorithm resulted in the detection and extraction of 16S rRNA gene sequences from the complete downloaded genomes, which were subsequently stored in FASTA-formatted files along with the variants, that is, the sequences differing by at least one nucleotide between each other in a genome. All the 16S rRNA gene variants identified were designated at the strain level or the species level if no designated strain name existed, in accordance with the established nomenclature guidelines.

A number of the genomes were found to lack 16S rRNA genes, resulting in the number of genomes reducing to 3, 192 oral-bacterial genomes, corresponding to 3, 047 strains and 334 species, and 191 oral-archaeal genomes, corresponding to 135 species. For the bacterial genomes, a total of 14, 966 genes and 8, 155 variants were identified. For the archaeal genomes, a total of 346 genes and 255 variants were identified.

For each genome under consideration, the following data were calculated: genome size, size of the 16S rRNA genes detected, total number of 16S rRNA genes, number of different variants, and number of 16S rRNA genes in each strand.

Additionally, through the use of a complementary script based on Python’s NumPy^43^ and pandas^44^ modules, the average, median, mode and standard deviation of the data obtained can be determined for hierarchical levels above the variant level.

## Data Records

The dataset is available at Zenodo (https://zenodo.org/records/15209015)^45^.

The 16SGOSeq dataset^45^ is provided in eight files, comprising both tabular and FASTA formats. The dataset comprises four files pertaining to bacteria and four files pertaining to archaea.

### Variants table (bacteria_variants.csv/.xlsx, archaea_variants.csv/.xlsx)

A single table is included in both the CSV and XLSX formats, containing all the variants identified in all the genomes. The file comprises as many rows as there are variants per group of GenBank identifiers (genome and plasmids). The pertinent data for each variant is included, such as the sequence, the number of copies, and the position of the variant in the genome, among other details (see Table 1).

**Table 1:** Description of the parameters associated with the variants CSV/XLSX file.

Parameter	Description
*gbid*	Genbank identifier or identifiers, and/or HOMD identifier
*taxonomy_id*	Taxonomy ID
*mean_genome_length*	Average length of the analysed genomes
*variant_length*	Average length of the variants found
*num_genes*	Number of genes (copies of each variant) found in the genomes
*num_genes_+strand*	Number of genes on positive strand
*num_genes_-strand*	Number of genes on negative strand
*positions*	Strand and positions, initial and end, where each sequence was found in the respective genome
*superkingdom*	Name of the superkingdom (bacteria or archaea)
*phylum*	Name of the phylum
*class*	Name of the class
*order*	Name of the order
*family*	Name of the family
*genus*	Name of the genus
*species*	Name of the species
*strain*	Name of the strain
*variant*	Variant identifier assigned
*sequence*	DNA sequence as it was found in the genome

### Variants FASTA (bacteria_variants.fasta, archaea_variants.fasta)

A FASTA file corresponding to the variants table contains one line per variant in each genome. The header includes the genome GenBank identifier, the full taxonomy up until the variant level, and the number of gene copies in the genome.

### Genes FASTA (bacteria_genes.fasta, archaea_genes.fasta)

A FASTA file is provided containing all sequences identified in all genomes. This file illustrates the copies of variants observed in the genomes, with each header including the genome GenBank identifier, the full taxonomy up to the variant level, as well as the positions of the genes in each genome and the strand in which it was found.

### Intragenomic variant divergence (bacteria_divergence.csv/.xlsx, archaea_divergence.csv/.xlsx)

A table is included in both the CSV and XLSX formats for bacteria and archaea, containing the information about the divergence existent between the variants of each genome of the dataset. It was acquired using BLASTN^46^ to align each genome’s variants against each other, to obtain the query coverage and the identity percentage used to evaluate the divergence.

Each genome has several rows corresponding to the alignments of each variant against the other variants of the genome. Table 2 shows the pertinent data included to assess the divergence, including the query coverage and the identity percentage.

**Table 2:** Description of the parameters associated with the intragenomic variant divergence CSV/XLSX file.

Parameter	Description
*gbid*	Genbank identifier or identifiers, and/or HOMD identifier
*qseqid*	Query sequence identifier
*sseqid*	Subject sequence identifier
*pident*	Identity percentage (percentage of identical matches)
*qcovs*	Query coverage percentage
*length*	Alignment length
*mismatch*	Number of mismatched bases
*gapopen*	Number of gap openings
*qstart*	Start position in the query sequence
*qend*	End position in the query sequence
*sstart*	Start position in the subject sequence
*send*	End position in the subject sequence
*evalue*	Expect value (E-value)
*bitscore*	Bit-score of the alignment

## Technical Validation

To validate the dataset, a total of 2, 039 random bacterial sequences were selected from our dataset, representing 25% of the total number of sequences. This random sequence group was aligned using BLASTN^46^ against a 16S rRNA gene sequence database. This smaller database includes 26, 954 sequences from bacteria and archaea, and does not necessarily include the same taxa represented in our dataset. With the alignment, we obtained an identity percentage of ≥97% in all cases, confirming that our sequences can be considered 16S rRNA gene sequences.

Additionally, we aligned with the same 25% of our bacterial dataset against the Core nucleotide database (*core_nt*)^35^, which contains 112, 880, 307 GenBank+EMBL+DDBJ+PDB+RefSeq sequences. In all cases, either the genus and species or the NCBI identifier matched between the query (our sequences) and the subject (*core_nt* sequences). Without exception, a query coverage of 100% and an identity percentage of ≥99% was obtained, confirming the validity of our sequences by demonstrating both their existence and their correct taxonomic annotation.

For the archaeal dataset, 64 sequences were selected, representing the 25% of the dataset. As done for the bacteria, all the sequences were aligned with BLASTN^46^ against the 16S rRNA gene sequence database from NCBI, obtaining an identity percentage of ≥97% again in all cases. These sequences were also aligned *core_nt*, with either the genus and species or the NCBI identifier matching between the query and the subject. A query coverage of 100% and an identity percentage of ≥99% were obtained.

Additionally, the intragenomic divergence of the variants was analysed. BLASTN^46^ was used to perform a discontinuous megablast and align each genome’s variants against each other. Genomes were considered to present high divergence if, at least two of their variants presented a query coverage of ≤97% or an identity percentage of ≤97%.

From the total 3, 046 bacterial genomes and 177 archaeal genomes, we have found 43 and 9, respectively, to present high divergence amongst some of their variants.

These results are presented in files bacteria_divergence.csv/.xlsx and archaea_divergence.csv/.xlsx in the dataset repository.

## Usage Notes

The quantity of PCR-amplified product is contingent upon the genome size and the number of 16S rRNA genes per genome^47^. Consequently, it is not possible to accurately quantify the number of species represented in clone libraries of samples from a given ecosystem until these two parameters are known for the taxa present^47^. If these factors are not considered, when performing PCR, qPCR, or marker-gene sequencing, inferences about numerous aspects of microbial communities may be affected^48^. Moreover, this information is also pertinent to whole genome sequencing (WGS) technologies, which employ 16S rRNA gene counts for their analyses of the diversity and structure of prokaryotic populations^49,50^. Understanding the number of gene copies per genome can facilitate our comprehension of the ecological and evolutionary relationships between different microorganisms^17^.

It can be observed that rrnDB^14^ and RiboGrove^15^ present certain inherent limitations when employed in studies of the mouth microbiome compared to 16SGOSeq^45^ (Table 3). The gene sequences included in our newly developed dataset originate from complete genomes that have been manually monitored at NCBI^36^ of bacteria and archaea that are known to inhabit the human oral cavity^34^. This enables researchers to ascertain the number of 16S genes and variants per genome at a desired hierarchical level or taxon. This information is necessary to adjust gene-based abundance values for estimated abundance values of the organism(s).

**Table 3:** Comparison of features between 16SGOSeq and other 16S rRNA gene copy number databases.

Feature	16SGOSeq	rrnDB	RiboGrove
Niche	Oral cavity	Multiple, unspecified	Multiple, unspecified
Supervised and curated data	*✓*		*✓*
Variant detection	*✓*		
FASTA containing the copies of the gene	*✓*	*✓*	*✓*
FASTA containing the variants of the gene	*✓*		
Statistic values calculation when filtered	*✓*	*✓*	
Filtering by taxonomy of the FASTA files	*✓*		*✓*
No additional bioinformatics processing required	*✓*		

The 16SGOSeq^45^ can be filtered according to the needs of the research study, grouping the genes by different taxonomic levels and obtaining the averages, standard deviations, and more calculations. Users can use programming languages such as R or Python, which are the most typically used in bioinformatics, to taxonomically filter the dataset using the columns in the dataset files dedicated to the taxonomy. Complementary, to facilitate the use of 16SGOSeq^45^, we constructed an auxiliary Python^51^ script to perform the filtering of the information by the desired taxonomic level and calculate averages of relevant data. Further details about the script can be found in the Supplementary Information.

The list of the oral bacteria included in our dataset originates from the eHOMD^25^. However, there is no database with sequences of archaea detected in the oral cavity. As there is no specific list of oral archaea in the eHOMD^25^, the complete genomes of the oral archaea were obtained directly from the NCBI^36^, and most of them belong to environmental niches. It is recommended that the oral microbiology community increase the amount of evidence related to oral archaea to overcome this limitation by focusing on isolating and sequencing archaea directly from mouth samples to refine taxonomic and functional annotations.

Most of the complete genomes in the eHOMD^25^ belong to cultivable taxa. While in the HOMD, 71% of the species are cultivable and 26% are non-cultivable, in the 16SGOSeq^45^ dataset 96% are cultivable and 1.20% are non-cultivable. Our dataset provides a more robust representation of the cultivable fraction of the oral microbiome than the non-cultivable. Nevertheless, 16SGOSeq^45^ contains the most prevalent and abundant bacterial species in health, caries, and periodontitis^1,2,52^, and numerous examples of the so-called rare taxa^53–55^.

So far, the sequences and data averages calculated in 16SGOSeq^45^ have been used in two studies by our research group. One to analyse the impact of intragenomic redundancy of the 16S rRNA gene and the selection of primer pairs in the oral cavity, and the other to identify species with very similar 16S rRNA sequence segments using different primer pairs^56,57^.

Like other applications, the 16SGOSeq^45^ can be used in the field of oral microbiology, facilitating the design of universal primers or probes capable of detecting the greatest possible diversity of oral prokaryotic organisms, and specific primers or probes that consider the sequences of all the genetic variants of a given taxon. Additionally, if sequences from 16SGOSeq^45^ are to be employed for the identification of primers or probes, we propose the utilisation of the PrimerEvalPy tool^58^. This is a Python^51^ application developed by our research team that performs a coverage analysis of any primer against any database. These advances enable more accurate detection and characterisation of oral microbiota and improve the understanding of the oral ecosystem and its role in health and disease. The 16SGOSeq^45^, with high-quality sequences and robust taxonomic annotations, can significantly refine our understanding of phylogenetic relationships among taxa.

The techniques and strategies implemented in the curation of the present dataset can be applied by clinical microbiologists, bioinformaticians or microbial ecologists in other microbiome fields. Our pipeline can be followed to generate gene copy number datasets from existing niche-specific datasets, ensuring the production of taxonomically robust, high-resolution, and biologically informative data.

## Supplementary information

16SGOSeq Supplementary Information

## Data Availability

The dataset and code can be accessed via Zenodo (https://zenodo.org/records/15209015)^45^ or the Gitlab repository at the following link: https://gitlab.citius.gal/lara.vazquez/16sgoseq. The dataset construction code was developed in Python 3.9, which was employed to generate the files composing the 16SGOSeq^45^ dataset. Furthermore, a complementary script is provided for the purpose of filtering and generating information from the dataset. Instructions for installing and using the script are detailed in the repository. 16SGOSeq^45^ follows an annual update policy, incorporating HOMD updates when available.
