# Supplementary material for: A curated bacterial and archaeal 16S rRNA Gene Oral Sequences dataset
Source: Sci Data. 2025 May 2;12:729. doi: 10.1038/s41597-025-05050-4 (PMC12048654; doi:10.1038/s41597-025-05050-4)
Supplement: Supplementary file 1 — 16SGOSeq Supplementary Information [file 41597_2025_5050_MOESM1_ESM.pdf]

16SGOSeq Supplementary Information

1 Supplementary Methods

1.1 Complementary Filtering Script

This script can generate a CSV file containing the average, median, mode and standard deviation per taxonomic group of the size of the genomes analysed and the genes and variants detected, the total number of different genes and variants, and the number of genes in each strand (positive and negative). Furthermore, it can filter the dataset by a specific taxon, for instance, the genus *Escherichia*. In this case, it can generate a FASTA file comprising the 16S rRNA gene sequences identified in the *Escherichia* genomes, as well as a CSV file containing the filtered dataset with the *Escherichia* variants.

In order to use the aforementioned complementary script, it is necessary to install Python 3.9 [1] or a later version. To execute it, the CSV input file containing the variants must be provided as the first parameter, and the desired taxonomy level/taxon as the second parameter. The path to the folder where the results are to be stored can be specified as an additional parameter. Additionally, a taxonomy abundance histogram can be generated if the dataset is filtered by a specific taxon. The generated plot is a histogram with one bar per taxonomic level, from superkingdom to variant, displaying the number of taxa present at each level (using the complete taxonomic path). The script can be executed and tested in the supplied Jupyter Notebook.

Further information can be accessed via Zenodo (`https://zenodo.org/records/15209015`) or the Gitlab repository at the following link: `https://gitlab.citius.gal/lara.vazquez/16sgoseq`

2 Supplementary Tables

Supplementary Table S1: Archaea NCBI accession identifiers downloadable from the NCBI nucleotide database.

NCBI Accession Number
CP002098.1
CP003083.1
CP003316.1
NC_000854.2
NC_000909.1

NCBI Accession Number
NC_000961.1
NC_002607.1
NC_002689.2
NC_003364.1
NC_003551.1
NC_003552.1
NC_003901.1
NC_005791.1
NC_005877.1
NC_007181.1
NC_007355.1
NC_007426.1
NC_007796.1
NC_007955.1
NC_008212.1
NC_008553.1
NC_008698.1
NC_008818.1
NC_008942.1
NC_009033.1
NC_009051.1
NC_009135.1
NC_009376.1
NC_009464.1
NC_009515.1
NC_009634.1
NC_009635.1
NC_009637.1
NC_009712.1
NC_009776.1
NC_009975.1
NC_011529.1
NC_011766.1

NCBI Accession Number
NC_011832.1
NC_012028.1-NC_012029.1
NC_012588.1
NC_012589.1
NC_012622.1
NC_012623.1
NC_012632.1
NC_012726.1
NC_012804.1
NC_012883.1
NC_013156.1
NC_013158.1
NC_013201.1-NC_013202.1
NC_013407.1
NC_013665.1
NC_013741.1
NC_013743.1
NC_013769.1
NC_013790.1
NC_013849.1
NC_013887.1
NC_013922.1-NC_013923.1
NC_013967.1
NC_014002.1
NC_014122.1
NC_014205.1
NC_014222.1
NC_014253.1
NC_014297.1
NC_014374.1
NC_014408.1
NC_014507.1
NC_014537.1

**NCBI Accession Number**
NC_014658.1
NC_014729.1
NC_014804.1
NC_014961.1
NC_015216.1
NC_015315.1
NC_015320.1
NC_015416.1
NC_015435.1
NC_015474.1
NC_015518.1
NC_015562.1
NC_015574.1
NC_015636.1
NC_015666.1
NC_015676.1
NC_015680.1
NC_015847.1
NC_015865.1
NC_015931.1
NC_015943.1-NC_015948.1
NC_016070.1
NC_016645.1
NC_017034.1
NC_017275.1
NC_017276.1
NC_017459.1
NC_017461.1
NC_017527.1
NC_017946.1
NC_017954.1
NC_018001.1
NC_018015.1

**NCBI Accession Number**
NC_018224.1
NC_018227.2
NC_018719.1
NC_019791.1
NC_019792.1
NC_019943.1
NC_019962.1
NC_019964.1
NC_019974.1
NC_019977.1
NC_020246.1
NC_020247.1
NC_020388.1
NC_020913.1
NC_021058.1
NC_021169.1
NC_021353.1
NC_021355.1
NC_021592.1
NC_021921.1
NC_022084.1
NC_022521.1
NC_023010.2-NC_023013.1
NZ_AP011526.1
NZ_AP011528.1
NZ_CP006019.1
NZ_CP006933.1-NZ_LN734822.1
NZ_CP006965.1
NZ_CP007055.1
NZ_CP007060.1
NZ_CP007174.1
NZ_CP007536.1
NZ_CP008746.1

NCBI Accession Number
NZ_CP008887.1
NZ_CP009149.1
NZ_CP009501.1
NZ_CP009502.1
NZ_CP009503.1
NZ_CP009504.1
NZ_CP009505.1
NZ_CP009506.1
NZ_CP009507.1
NZ_CP009508.1
NZ_CP009509.1
NZ_CP009511.1
NZ_CP009512.1
NZ_CP009513.1
NZ_CP009514.1
NZ_CP009515.1
NZ_CP009516.1
NZ_CP009517.1
NZ_CP009518.1
NZ_CP009520.1
NZ_CP009524.1
NZ_CP009526.1
NZ_CP009528.1
NZ_CP009530.1
NZ_CP010070.1
NZ_CP010529.1
NZ_CP011266.1
NZ_CP013050.1
NZ_CP017686.1
NZ_CP017803.1
NZ_CP020120.1-NZ_CP026606.1
NZ_CP020360.1-NZ_CP020361.1-NZ_CP020362.1-NZ_CP020363.1-NZ_CP020364.1
NZ_CP039136.1-NZ_CP039138.1

NCBI Accession Number
NZ_CP039375.1-NZ_CP039376.1
NZ_CP042908.1
NZ_CP045142.1
NZ_CP048739.1-NZ_CP050274.1
NZ_LR698975.1
NZ_LT158599.1
NZ_LT607756.1

References

[1] Python Software Foundation Python. (https://www.python.org/,2020), Accessed: 2024-05-22
